# Effect of agarose/gelatin gel addition on the pro-angiogenic potential of polyhydroxybutyrate/chitosan scaffolds

**DOI:** 10.3389/fcell.2024.1504268

**Published:** 2025-01-21

**Authors:** Mária Giretová, Ľubomír Medvecký, Zuzana Demčišáková, Lenka Luptáková, Eva Petrovová, Radoslava Štulajterová

**Affiliations:** ^1^ Division of Functional and Hybrid Materials, Institute of Materials Research of SAS, Košice, Slovakia; ^2^ Department of Morphological Disciplines, University of Veterinary Medicine and Pharmacy in Košice, Košice, Slovakia; ^3^ Department of Biology and Physiology, University of Veterinary Medicine and Pharmacy in Košice, Košice, Slovakia

**Keywords:** gel, polymeric scaffold, quail embryo CAM assay, angiogenesis, cytotoxicity

## Abstract

The aim of this paper was to evaluate the effect of gel addition to biopolymeric scaffolds on the pro-angiogenic and basic material characteristics of the final composite for use in regenerative medicine. The studied scaffold consisted of natural biopolymers: polyhydroxybutyrate, chitosan, agarose, and gelatin. The final scaffold was characterized by high macroporosity (90%) and wide pore size distribution. As is known, the pore size is a critical factor for cell ingrowth in grafts after implantation in the body and for angiogenic development and creation of new vessels. After 9 days of cultivation in the culture medium, the scaffold retained its physicochemical properties without any tendency of disintegration. The addition of polymeric gels to the scaffold improved the mechanical stability of the composite. *In vitro* cytotoxicity testing showed good adherence of the seeded L929 fibroblasts on the scaffold and strong ingrowth of cells into the macropores. No sign of cytotoxicity was identified by both the MTS assay and live/dead cell staining. The quail chorioallantoic membrane (CAM) assay—as an alternative to *in vivo* assays—revealed suitable pro-angiogenic properties of the scaffold for the formation and ingrowth of new blood vessels. Moreover, the upregulation of gene expression responsible for the activation of angiogenic cascade clearly demonstrated a positive effect of the prepared composites on angiogenesis as an essential part of new tissue formation and the regeneration process itself.

## 1 Introduction

The aim of the interdisciplinary field of tissue engineering is to regenerate living tissues by replacing lost or damaged tissue or organs with appropriate scaffolds to recover organ function. This task requires an appropriate tissue engineering scaffold (Noro et al., 2024). Scaffolds should establish a favorable environment for the integration and attachment of cells or growth factors for supporting healing of various anatomical defects, diseases, and injuries of complex organs and tissues without toxic effects or inducing an inflammatory reaction (Rahmati et al., 2023).

Polysaccharides represented by chitosan are an important group of biopolymers due to their similarity to glycosaminoglycans, which is a part of the cartilage matrix of articular cartilage tissue. Chitosan is a biocompatible and biodegradable natural polymer composed of glucosamine and N-acetyl-glucosamine units and is soluble in dilute acids (Felfel et al., 2019). In addition to good biocompatibility, chitosan also has excellent antibacterial properties because the positively charged chitosan amino groups interact electrostatically with the negatively charged components of the outer membrane of bacteria and disrupt the bacterial cell wall, bond to microbial DNA, and cause the chelation of nutrients on the cell wall, which are essential for bacterial growth (Yan et al., 2021). Chitosan-based coatings and materials have been applied in tissue engineering scaffolds for vascular regeneration (Wang et al., 2023). It shows coagulation-promoting abilities, making it a traditional biomaterial for wound dressing (Li et al., 2021; Jung Bingjun and Zhao, 2015). Utilization of chitosan and chitosan-based nanocomposite as drug delivery systems was described in detail by Elgadir et al. (2015) and Raza et al. (2020).

Polyhydroxybutyrate is an aliphatic polyester, a member of a large family of polyhydroxyalkanoates (PHAs) produced by bacteria (Popa et al., 2022). PHAs have been extensively studied for tissue engineering and drug delivery applications due to their appropriate material properties like the mechanical properties similar to polypropylene and the ability of being almost completely degraded by microorganisms. PHAs are frequently used as a very suitable scaffold material supporting the growth and proliferation of cells and have been used for different biomedical applications (sutures, repair devices and patches, stents, articular cartilage, bone marrow scaffolds, etc.) (Mohandas et al., 2021). Poly-3-hydroxybutyrate (PHB) is a hydrophobic biopolymer that biodegrades hydrolytically without any toxic products. One product of its hydrolytic degradation is 3-hydroxybutyric acid, which is a normal constituent of blood. PHB has been extensively used in tissue engineering of bone, cartilage, tendon, skin, and nerves, predominantly in long-term tissue engineering applications (Guo et al., 2022; Mohan et al., 2021; Giretova et al., 2016). A blend of chitosan and PHB (PCH) induced *in vitro* formation of hyaline cartilage components. Preliminary *in vivo* experiments with acellular scaffolds, which filled the artificially created cartilage defect in the sheep knee, were also evaluated. Cells released from the bone marrow cavity penetrated into the acellular PCH scaffold in cartilage defect and induced tissue formation similar to the hyaline cartilage (Giretova et al., 2019).

Agarose (AG) is a polysaccharide derived from marine red algae, consisting of D-galactose and 3,6-anhydrous-l-galactose units. Agarose in the form of beads manifested highly porous, mechanically resistant, chemically and physically inert, and hydrophilic properties (Schindler et al., 2021; Zucca et al., 2016). In blends with other polymers or calcium phosphates, it is utilized in nervous system regeneration, bone and cartilage tissue engineering, and as a skin substitute (Bhat and Kumar, 2012; Felfel et al., 2019; Khanmohammadi Garakani et al., 2020; Kazimierczak et al., 2019; Sivashankari and Prabaharan, 2020). The subcutaneous AG implants induced new vessels and fibrous tissue formation; the capsule thickness around AG remained thinner than those around hyaluronic acid and collagen (Varoni et al., 2012). In addition, the artificial skin substitute in the form of chitosan/agarose film expressed an acidic pH of 5.98, favorably affecting skin regeneration, with biodegradable properties in a simulated enzymatic wound environment. The film was capable of high elastic deformation in the wet state, which allowed stretching in wound application (Vivcharenko et al., 2020).

Gelatin is a natural protein derived from collagen by hydrolysis. Compared to collagen, gelatin offers improved viscosity, elasticity, and conductivity; exhibits lower toxicity and acceptable mechanical strength for bioscaffold preparation and similar bioactivity to collagen; and minimizes inflammation and an adverse reaction of the organism after implantation (Asl et al., 2024).

The gelatin–chitosan scaffold doped with Mg–Fe showed great potential for clinical application in nerve regeneration in *in vivo* tests (Zhang et al., 2023). Rashid et al. (2023), in his review work, described a broad spectrum of published research works that deal with the successful usage of gelatin-based scaffolds in skin, cartilage, bone, liver, and cardiovascular tissue engineering applications (Rashid et al., 2023).

The highly vascularized avian chorioallantoic membrane (CAM) was widely used as an alternative *in vivo* model to study angiogenesis before more extensive *in vivo* studies in mammals and has become an integral part of the biocompatibility testing of biomaterials for regenerative medicine (Schneider-Stock and Ribatti, 2021). In 1989, Spanel-Borowski first described the use of the CAM in the implantation and biocompatibility testing of biomaterials (Spanel-Borowski, 1989). The exploitation of the avian CAM model respects the 3R principles (replacement, reduction, and refinement) for the welfare and ethics related to the use of animal mammalian experimental models in product testing including biomaterials and scientific research. The CAM assay is a simple, rapid, and cost-effective model for screening a large number of samples for various applications within a short timeframe (Ribatti, 2016). Since the CAM is not innervated and experiments are terminated before the development of pain centers in the brain, ethical concerns associated with animal experimentation are minimized. As the chick embryo is naturally immunodeficient, it can accept transplants from diverse tissues and species without eliciting an immune response. By placing specimens on the CAM, researchers can directly observe and analyze real-time changes in morphology and the development of new vessels. In mammalian *in vivo* assays, complex surgery to place implants is needed, and the visualization of an implant or test site is usually not visible to the naked eye. The implantation itself and the test time are longer. Physiology and biology of mammalian models are also well known, but they are also much more complex. CAM assay is easily reproducible in relation to mammalian models, for which reproducibility is expensive and requires more time and labor (Tufan and Satiroglu-Tufan, 2005). Existing data suggest that the CAM assay can serve as a valuable pre-clinical screening tool to assess the biocompatibility of scaffolds and evaluate their potential for tissue engineering applications (Ribatti et al., 2020). The CAM model serves as an intermediate step between simple *in vitro* models and complex *in vivo* animal models for testing biomaterials. By reducing the need for *in vivo* animal studies and rapid biocompatibility screening, it contributes to the advancement of regenerative medicine (Petrovova et al., 2019).

The CAM alternative model successfully used a quail (*Coturnix japonica*) embryo as an alternative to the more widely used chick (*Gallus gallus*) embryo. During the first 5 days of incubation, the developmental stages of both species are identical. At quail ED8–8.5 (which corresponds to the chick ED8–9) and from this point to the later stages, the quail embryonal development is more rapid (Kundeková et al., 2021). The thinner quail shell is easy to open, and its cultivation and manipulation are simpler compared to that of the chicken embryos. The smaller quail embryos could be incubated in smaller containers (6-well cultivation plates), which would save space in the incubator (Čavarga et al., 2014). Angiogenesis plays an important role in tissue repair and wound healing. Development of blood vessels is a crucial step in regenerative medicine because the lack of active vascular ingrowth leads to transplant rejection due to necrosis in the depth of the tissue-engineered grafts (Jahani et al., 2020; Mangir et al., 2019; Demcisakova et al., 2022). The pro-angiogenic ability of grafts is manifested by the formation of new blood vessels from pre-existing vessels on the implantation site of the chorioallantoic membrane (Tufan and Satiroglu-Tufan, 2005).

The cascade of processes of angiogenesis is controlled by the multiple growth factors, endocrine and paracrine molecules. Among these factors, the vascular endothelial growth factor (VEGF), fibroblast growth factor-2 (FGF-2), angiopoietins (ANG-1 and ANG-2), and vascular endothelial cadherin (VE-CAD) are of the highest importance (Carmeliet and Jain, 2011). Hypoxia of the tissues plays a crucial role in initiating angiogenesis. It is responsible for the start of the production of VEGFA in hypoxic tissue, which triggers the migration of the endothelial cells and the differentiation of endothelial cells into tip cells and stalk cells, which are involved in the sprout initiation and activation of VEGFR2 on endothelial cells (Abhinand et al., 2016). VE cadherin is involved in the formation of adhesive junctions between endothelial cells. After stimulation by VEGFA, VEGFR2 binds to VE-cadherin and Src kinase, and the adhesive junctions are disrupted. Therefore, a decrease in VE-cadherin can be observed during angiogenesis (Nan et al., 2023). Angiopoietin 2, in the presence of VEGFA, is responsible for the endothelial cells’ proliferation, their migration and sprouting, and promotion of growth of new blood vessels; therefore, it serves as a pro-angiogenic molecule (Asahara et al., 1998).

In the paper, we studied the effect of gel addition to the chitosan/PHB scaffold on the final pro-angiogenic potential of the composite. The composition and cohesion between chitosan/PHB fibers or particles in the scaffold were modified by application of agarose/gelatin gel on the lyophilized scaffold, which led to the formation of a mechanically stable and highly porous (porosity 90%) microstructure for good handling, adherence, and proliferation of the mouse fibroblastic cell line L929 in *in vitro* conditions and for using in the alternative CAM model to analyze pro-angiogenesis stimulating properties.

## 2 Materials and methods

### 2.1 Preparation of composites and biopolymer scaffolds

PHB (Goodfellow, Cambridge, England)/chitosan scaffolds (low molecular weight, Sigma-Aldrich, St. Louis, MO, United States) with a weight ratio equal to 1:1 were prepared by mixing PHB (dissolved in propylene carbonate (at 130°C, 2% (w/v) solution) and chitosan solutions (in 1% acetic acid, 1% (w/v) solution). Mixing was carried out using a magnetic stirrer at 400 rpm. The biopolymers were precipitated from the mixture by the addition of acetone (Sigma-Aldrich, for analysis) and NH_3_ (aq, 25%, Fluka). The final suspensions were filtered, washed with distilled water, and loaded into a polypropylene syringe (50 mL) to form disc-shaped samples (4 cm in diameter and 5 mm in height). The samples were frozen at −20°C and lyophilized (lyophilizer, ilShin Biobase Europe, Ede, Netherlands) for 8 h.

The evaluation of water uptake by the prepared scaffolds was carried out by immersing the substrates (approx. 100 mg) in 0.9% NaCl solution at 37°C until a constant mass of samples was achieved. Soaking was carried out three times, and the water uptake was calculated as the ratio of the weight of the wet sample to the weight of the original dry sample. The calculated value was equal to 5.2 and was used to select the appropriate volume of gel to be added to the scaffold surface for the preparation of scaffold/gel composites. The open porosity of the PHB/chitosan scaffolds was determined by filling the pores with ethanol (Sigma-Aldrich, absolute, HPLC grade) after exhausting the air from scaffolds using a vacuum pump. Porosity was calculated as the ratio of the volume of ethanol in the pores (obtained from the weight changes between the dry and wet samples and the density of 100% ethanol) and the volume of the dry sample measured from the sample dimensions.

**FIGURE 1:**
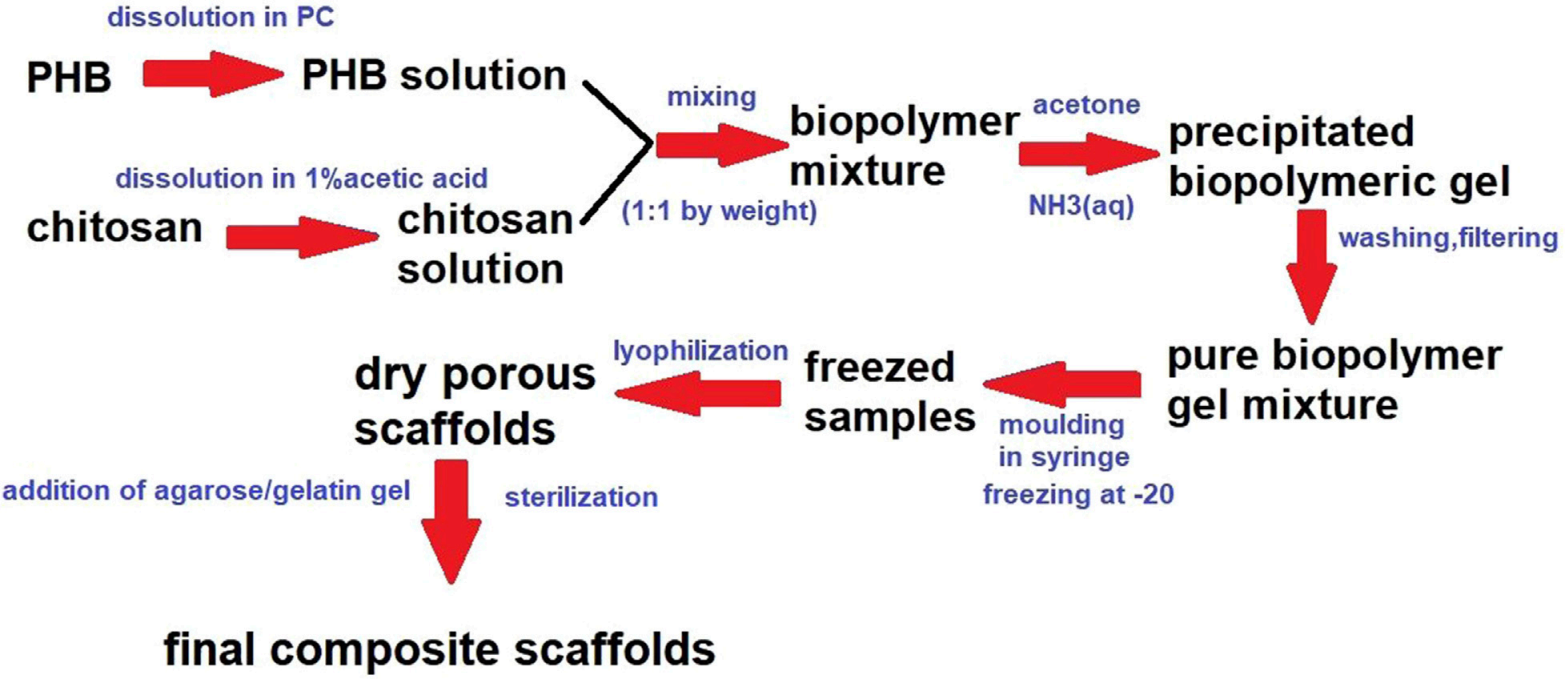
Biopolymer preparation scheme.

Scaffold/gel composites (AG-PCHLY) were prepared by adding a warm gel containing 2% (w/v) agarose and 1% (w/v) gelatin to the dry scaffold surface. The final biopolymer composite scaffolds were sterilized in an autoclave at 121°C. Figure 1 shows the scheme presenting the preparation of biopolymeric scaffolds.

### 2.2 Characterization of porosity, the average molecular weight of chitosan and PHB, and FTIR analysis of composites

The microstructure and pore size distribution of the prepared samples were observed with an inverted optical fluorescence microscope (Leica DM IL LED, Heerbrugg, Switzerland) equipped with a CCD camera in the VIS mode at ×50 magnification. The pore size distribution was calculated from 2-mm^2^ surface area (five different locations) using ImageJ8 software for image analysis. Pore sizes were divided into five pore size fractions.

The average molecular weight (M_w_) of chitosan and PHB was determined by gel permeation chromatography (GPC, Watrex, Prague, Czech Republic, RI detector). Chitosan separation was performed on a PL gel-mixed OH 8-μm column at a mobile phase flow rate of 1 mL/min (mobile phase 0.1 M NaH_2_PO_4_, pH = 6) with a UV-vis detector (SYKAM S3240) at 230 nm. For PHB, separation was performed on a PL gel-mixed C 5-mm column with chloroform as the mobile phase and an RI detector (RI 2000, Schambeck SFD, Bad Honnef, Germany). The average molecular weights of chitosan and PHB were determined from calibration curves using dextran (chitosan) and polystyrene (PHB) as the standards with different average molecular weights and calculated by Clarity datastation software.

The mutual interaction and crystallinity of biopolymers were evaluated by FTIR spectroscopy (IRAffinity-1, Shimadzu, Kyoto, Japan, KBr method).

### 2.3 Cytotoxicity test

The L929 mouse fibroblasts (ECACC, Salisbury, UK) were used for *in vitro* determination of cytotoxicity. Cells were cultured in culture flasks with 75-cm^2^ surface areas in MEM (minimum essential medium Eagles) culture medium with the addition of 10% fetal bovine serum and ATB-antimycotic (penicillin, streptomycin, and amphotericin) solution (all from Sigma-Aldrich, St. Louis, MO, United States). Cells were maintained at 37°C in a 5% CO_2_ atmosphere with 95% humidity in an incubator (Memmert, Schwabach, Germany). The medium was changed every 2 days. After the cells reached approximately 80% confluence, they were harvested by trypsinization using 0.25% trypsin–EDTA (Sigma-Aldrich) solution, followed by the addition of fresh medium to create a cell suspension, and the cell numbers were calculated using a Neubauer hemocytometer.

Solid wet samples (PCHLY, AG-PCHLY) for *in vitro* testing were cut with a corkscrew from the prepared sterilized biopolymeric foils. The sterile samples (Ø6 mm, height 2 mm) were placed in wells of a 48-well cell culture plate (Sarstedt, Numbrecht, Germany) and seeded with 2.0 × 10^4^ cells in 500 μL (4.0 × 10^4^ cells/mL) of complete medium and cultured in an incubator. Cell proliferation was examined using an MTS test (cell titer 96 aqueous one-solution cell proliferation assay, Promega, Madison, United States) 48 h and 9 days after cell seeding. Immediately before the assay, the culture medium was removed from the wells and replaced with fresh medium, and the MTS reagent was added. After 3 h of incubation, the intensities of coloring, which characterize the formazan concentration (produced by proliferating cells) in the culture medium, were evaluated using a UV-vis spectrophotometer (Shimadzu, UV-vis 1800, Kyoto, Japan) at a wavelength of 490 nm. The measured absorbances of the medium from the wells with cell-seeded substrates were compared with those from wells with seeded cells free of scaffolds.

### 2.4 Live/dead staining

The morphology, density, and distribution of cells on polymeric samples were determined by live/dead cell staining. Fluorescein diacetate reacts with enzymes of live healthy cells and is converted to a green fluorescent product; propidium iodide passes the damaged membranes of dead cells and stains the dead cells red. The cells on substrates were observed using a fluorescence microscope (Leica DM IL LED, blue filter) to investigate the morphology and distribution of cells 9 days post-seeding.

### 2.5 *In vivo* CAM model

#### 2.5.1 CAM *ex ovo* model for evaluation of the biocompatibility and angiogenic response to biomaterials

Fertilized quail eggs (*Coturnix coturnix japonica*, n = 158) were purchased and delivered from a quail farm (Mala Ida, Kosice, Slovakia). The eggs were cleansed with 70% ethanol and incubated horizontally in a forced-draft incubator at 38.2°C ± 0.5°C and 58% relative humidity (RH). After 56 h of incubation, each egg was cracked, and the quail embryo with egg content was carefully transferred into a sterile 6-well cell culture plate (TPP, Trasadingen, Switzerland). The embryos were incubated until ED6 in a still-draft incubator (38.2°C ± 0.5°C, 60% RH).

On ED6, the sterilized PCHLY and AG-PCHLY scaffolds (4 × 4 mm) were carefully placed on the CAM surface. After 72 h following the implantation (ED9), the CAM-material complexes were excised with the surrounding CAM tissue that remained around the scaffold (2 mm) for subsequent histological and immunohistochemical analyses. For molecular analysis, CAMs were excised without any border restrictions on ED11. Since it was not possible to isolate DNA from the samples collected 72 h after implantation, the samples for RT-PCR analysis were obtained 5 days after implantation.

Because the avian embryo is exempted from the European horizontal legislation on the protection of animals used for scientific purposes (EU Directive 2010/63/EU for animal experiments), ethical approval is not required.

#### 2.5.2 Histological examination

Histological examination of the CAM tissue reaction to biomaterials was performed from the complex of PCHLY or the AG-PCHLY scaffold and the surrounding CAM tissue. After 24-h fixation in Dent’s solution (a solution of methanol and dimethyl sulfoxide in a 4:1 ratio; Sigma-Aldrich, St. Louis, MO, United States), the samples were dehydrated in an ethanol series and embedded in paraffin. The specimens were serially cut into 7-μm sections using a rotary microtome (Leica RM 2244), deparaffinized, and hydrated with distilled water, followed by staining in the Alcian blue solution (Sigma-Aldrich, St. Louis, MO, United States). Then, nuclei were counterstained in Mayer’s hemalum solution (Millipore Sigma, St. Louis, MO, United States). Subsequently, sections were rinsed and stained with eosin (Sigma-Aldrich, St. Louis, MO, United States). Samples were dehydrated in an ethanol series and mounted in a permanent histological medium (Entellan, Millipore Sigma, St. Louis, MO, United States). All stained samples were evaluated using a biological microscope Olympus CX43 (Olympus, Tokyo, Japan) with a built-in digital camera (PROMICAM 3-5CP+; Promicra, Prague, Czech Republic) at ×10 magnification.

#### 2.5.3 Immunohistochemical analysis

The formation of blood vessels in the surrounding area of the PCHLY and AG-PCHLY scaffold and cell invasion into the tested implants were observed and evaluated using the markers of endothelial cells and hemangioblasts (quail endothelial cell surface marker; QH1), myofibroblasts (alpha smooth muscle actin, α-SMA), and the proliferative activity of the cells (phospho-histone H3, PHH3) with immunohistochemical staining.

Deparaffinized and rehydrated paraffin sections of the CAM with the scaffold were washed in PBS and PBS with 0.5% Tween 20 (Sigma-Aldrich, St. Louis, MO, United States) and then blocked in a normal goat serum (NGS, 1:10, Sigma-Aldrich, St. Louis, MO, United States) and 1% bovine serum albumin (BSA, Sigma-Aldrich, St. Louis, MO, United States) in 0.1% Triton-X (Sigma-Aldrich, St. Louis, MO, United States) for 60 min at room temperature (RT). Sections were then incubated overnight at +4°C with monoclonal mouse antibodies (QH1 1:1,000, Developmental Studies Hybridoma Bank, Iowa, United States; α-SMA 1:800, Sigma-Aldrich, St. Louis, MO, United States; PHH3 1:100, anti-phospho-histone H3 (Ser10) antibody, Mitosis Marker, Millipore, CA, United States). The sections were washed in PBS and incubated with appropriate secondary antibodies (1:200, Rhodamine AffiniPure Goat Anti-Mouse or Goat Anti-Rabbit IgG antibody, Jackson ImmunoResearch Laboratory, West Grove, PA, United States) in the dark at RT. Nuclei were counterstained with HOECHST (1:100,000, diluted in 0.1% Triton-X in distilled water, Sigma-Aldrich, St. Louis, MO, United States) for 10 min. The sections were washed in distilled water, dehydrated in an ethanol series, and mounted in Vectashield medium (Vectashield Antifade Mounting Medium, Vector Laboratories Inc., United States).

Fluorescent-stained paraffin sections of the quail CAM with implanted scaffolds were examined and documented using an Olympus BX53 fluorescence microscope (Olympus, Tokyo, Japan) and an Olympus DP74 digital camera.

#### 2.5.4 Macroscopic evaluation of the angiogenic response

The macroscopic evaluation of the angiogenic response was performed using the vascular index, which was measured as the difference between the number of blood vessels in the surrounding area of the implants at the beginning of treatment (the day of implantation, ED6) and the number of vessels 72 h after scaffold implantation (ED9). Newly formed CAM blood vessels were observed using an Olympus SZ61 stereomicroscope (Olympus, Tokyo, Japan) with a digital camera (PROMICAM 3-3CP; software QuickPHOTO MICRO 3.2, Prague, Czech Republic). A single macroscopic image was taken from each biological replicate (PCHLY: n = 10, AG-PCHLY: n = 8). The vessel counts were performed using ImageJ software Cell Counter Plugin (ImageJ 1.53e, United States). Images were first converted to grayscale (8-bit) and sharpened, and all vessels growing toward the scaffold were counted manually. All experimental procedures were repeated three times.

#### 2.5.5 Gene expression

The biomaterial was collected from CAM using scissors for molecular analysis on ED11. The analysis was conducted as previously reported (Luptakova et al., 2021). Total RNA was extracted from the biomaterial using QIAshredder and a total RNeasy Mini Kit (QIAGEN, Hilden, Germany), following the manufacturer’s instructions, including genomic DNA digestion using the RNase-free DNase set (QIAGEN, Germantown, TN, United States). The RNA purity and yields were analyzed using the NanoDrop Lite Spectrophotometer (Thermo Fisher Scientific, Waltham, MA, United States). We used a two-step RT-qPCR approach. In the first step, complementary DNA (cDNA) synthesis was performed using a protocol for the RT2 First Strand Kit (QIAGEN, Germany). A measure of 1 µg of total RNA was used to prepare 20 µL of cDNA, which was then used for qPCR. In the second step, the quantification of genes of interest in the cDNA samples was performed using specific primers for VE-cadherin (CDH5), angiopoietin-2 (ANGT2), vascular endothelial growth factor (VEGFA), and vascular endothelial growth factor receptor 2 (VEGFR2) (Table 1) (Macajova et al., 2020; Gheorghescu and Thompson, 2016). For each gene, a total volume of 25 µL of SYBR Green Master Mix (QIAGEN, United States) was used. The PCR mixture contained specific primers for each gene (300 nM), SYBR Green PCR Master Mix, and water. cDNA for GAPDH was used as an endogenous control for calculating fold differences in RNA levels by the 2^−ΔΔCT^ method. qPCR was performed under the same conditions for SYBR Green with the following steps: initialization at 95°C for 10 min and amplification in 40 cycles at 95°C for 15 s, followed by 60°C for 1 min. Dissociation curve analysis was performed after each completed PCR run to ensure the absence of nonspecific amplifications. The gene expression data were calculated against the GAPDH endogenous control, and the expression levels of selected genes were normalized to the untreated samples (control).

**TABLE 1:** Primers of genes for RT-PCR analysis.

Gene		Primer	Reference
Vascular endothelial growth factor receptor 2	VEGFR2 F	CAT​CAA​TGC​GAA​TCA​TAC​AGT​TAA​G	Macajova et al. (2020)
	VEGFR2 R	CAT​TCA​CAA​GCA​GGG​TGA​ATG	Macajova et al. (2020)
Vascular endothelial growth factor A	VEGFA F	CGG​AAG​CCC​AAT​GAA​GTT​ATC	Macajova et al. (2020)
	VEGFA R	GCA​CAT​CTC​ATC​AGA​GGC​ACA​C	Macajova et al. (2020)
Glyceraldehyde 3-phosphate dehydrogenase	GAPDH F	GAA​CGC​CAT​CAC​TAT​CTT​CCA​G	Macajova et al. (2020)
	GAPDH R	GAA​CGC​CAT​CAC​TAT​CTT​CCA	Macajova et al. (2020)
Angiopoietin 2	ANGT2 F	CAG​TGT​CTC​AAG​CGT​TCT​C	Gheorghescu and Thompson (2016)
	ANGT2 R	CTG​TCA​GAG​GAA​GGG​CAA​AG	Gheorghescu and Thompson (2016)
VE-cadherin	CDH5 F	TCT​GTC​CCA​GAA​ATG​TCA​CG	Gheorghescu and Thompson (2016)
	CDH5 R	CAC​CGG​AGT​CAT​CAA​CTG​TG	Gheorghescu and Thompson (2016)

### 2.6 Statistical analyses

The statistical analysis was performed using one-way ANOVA with Šidák´s multiple comparison test and nonparametric Mann–Whitney test using GraphPad Prism 10 software. All measurements were reported as mean ± standard deviation (SD) of three independent experiments. The differences were considered significant at p < 0.0001 and p < 0.005.

## 3 Results

### 3.1 Characterization of scaffold porosity, the average molecular weight of biopolymers, and FTIR analysis of composites

GPC analysis of the PHB/chitosan scaffolds showed that the M_w_ values of PHB and chitosan were 80 and 46 kDa, respectively, and comparison with M_w_ of the native biopolymers (120 kDa PHB and 49 kDa chitosan) verified an insignificant decrease in the M_w_ value of chitosan that was contrary to the approximately 30% decrease in the case of PHB. Thus, the preparation procedure and the formation of the biopolymer mixture mainly influenced the length of the PHB chains. A decrease in the average molecular weight of PHB during the dissolution of PHB in propylene carbonate has been confirmed at temperatures above 120°C due to the process of degradation by the mechanism of random splitting of the polymer chains, where the presence of propylene carbonate in the system significantly reduced the activation energy of the process (McChalicher et al., 2010). Notably, stronger hydrolytic degradation or dehydration of PHB in the aqueous solution occurs only under strongly alkaline or strongly acidic conditions with the production of 3-hydroxybutyric and crotonic acids (Yu et al., 2005). Hydrolysis of chitosan in a solution depends on its acidity (pH < 3.5) and has been shown to be much slower in weak acids (like acetic acid) than in stronger and more dissociated acids (e.g., HCl). Furthermore, the presence of a higher NaCl content in the solution partially hinders the hydrolysis of chitosan in acetic acid due to the contraction and closure of the glycosidic chain against the action of acidic hydrogen ions (Kasaai et al., 2013; Chen et al., 2009). The conditions used during the precipitation of biopolymers were too soft for deeper degradation of chitosan chains in terms of pH and during the dissolution of chitosan at room temperature, and both processes—dissolution of polymers and mutual mixing of polymer solutions—took place in a short time (several minutes).

**FIGURE 2:**
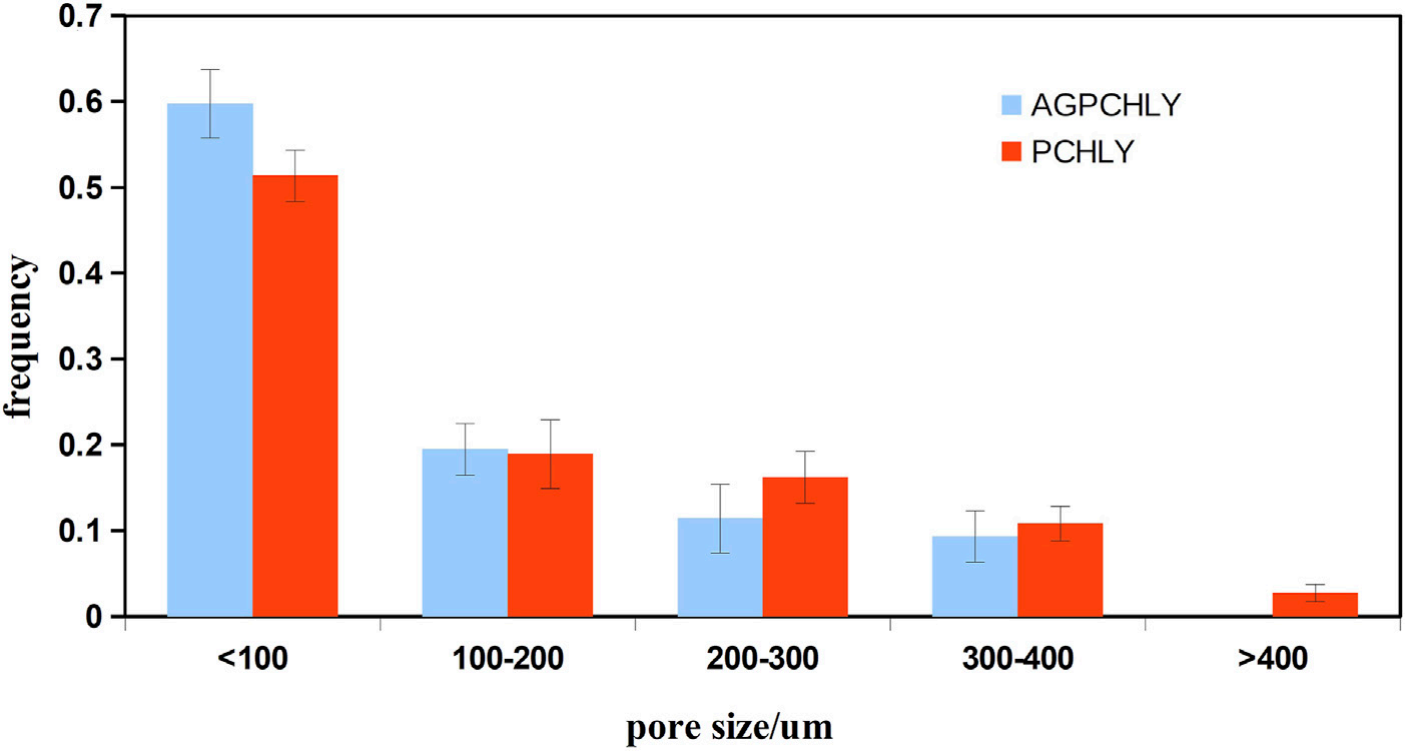
Pore size distribution of PHB/chitosan scaffolds (PCHLY) and scaffold/gel composites (AG-PCHLY).

The overall open porosity of the PHB/chitosan scaffolds was 90% ± 3% with a relatively broad pore size distribution, as shown in Figure 2.

**FIGURE 3:**
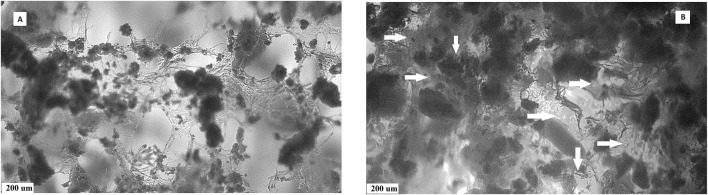
Microstructures of PCHLY **(A)** and AG-PCHLY **(B)** composite scaffolds (horizontal arrows: thickened amorphous gel structure-coated chitosan fibers; vertical arrows: gel shell around PHB aggregates).

A comparison of the pore size distribution revealed the disappearance or reduction in the size of larger macropores (>400 µm) and a small increase in the number of pores measuring <100 µm in size. This decrease in pore size could be caused by the pores getting filled with a relatively viscous gel. To verify this, macroscopic images of the microstructures of PCHLY and AG-PCHLY samples taken using an optical microscope were examined, which are shown in Figure 3. In the microstructure of PHB/chitosan scaffolds (Figure 3A), two different types of polymer structures can be identified: a fibrous network composed of transparent chitosan fibers that are often joined to the shape of plate-like structures, which form macropore walls and are responsible for the sponge-like microstructure; and irregularly shaped thick aggregates of PHB that are up to 400 um in size and are formed by the aggregation of several globular agglomerates consisting of PHB microparticles that were entrapped in the chitosan network during precipitation. On the other hand, the fibrous nature of the chitosan network was partially disordered (overlaid) by the agarose/gelatin gel due to coating of all structural polymeric objects—the chitosan structures (regardless of fibers or plate-like objects) and PHB aggregates (Figure 3B). The above facts indicate a decrease in open porosity of AGPCHLY scaffolds as a result of pore filling and possible swelling of chitosan fibers during autoclave sterilization. Note that under such conditions, including the soft spongy microstructure of the substrates, it is difficult to measure the correct value of this parameter, but the addition of gel to the PCHLY scaffolds significantly improved the strength of the scaffolds and allowed easier handling without the risk of damaging them.

**FIGURE 4:**
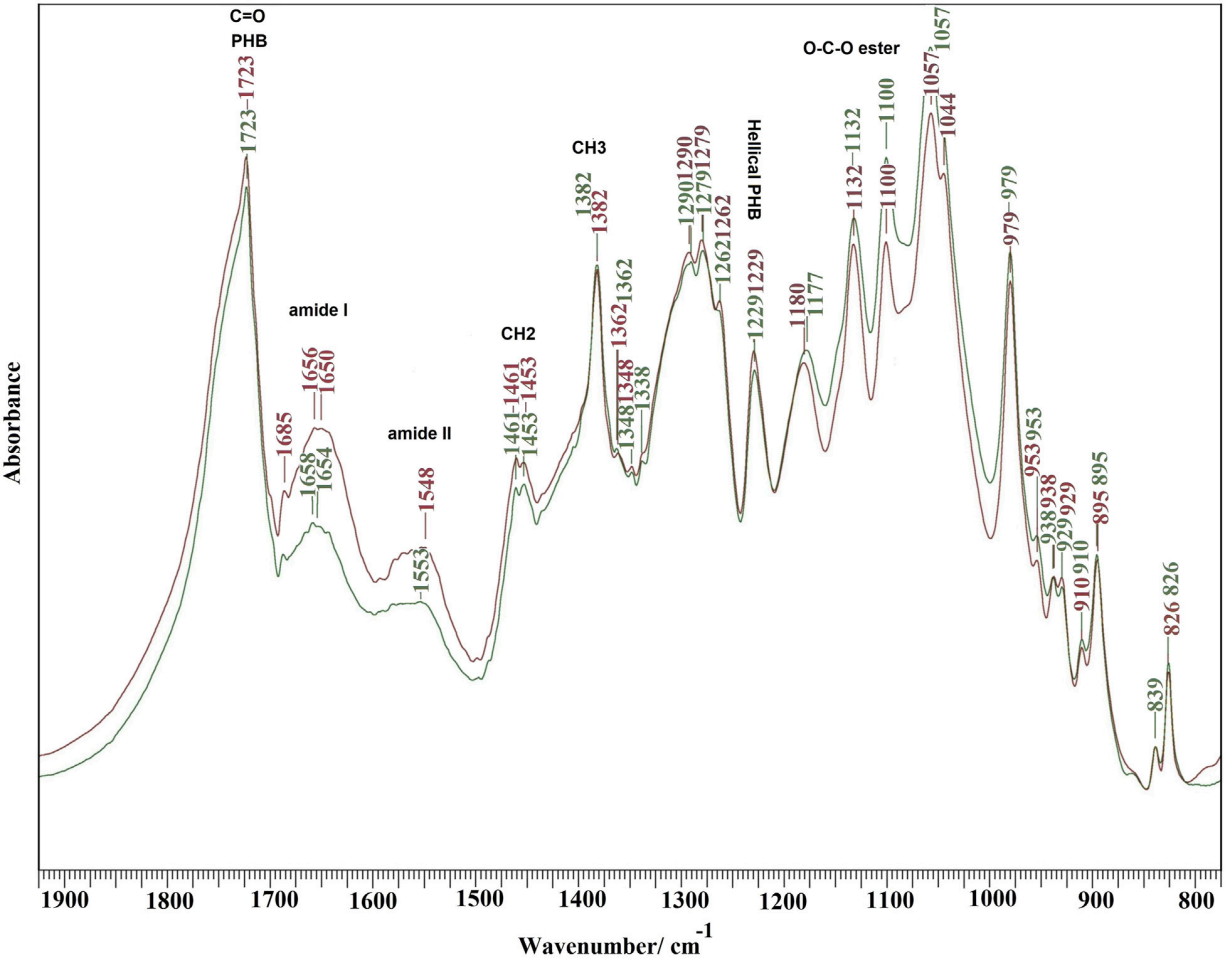
FTIR spectra of PCHLY and AG-PCHLY scaffolds.

FTIR spectra of PCHLY and AG-PCHLY scaffolds are shown in Figure 4. Very intense PHB bands from stretching vibrations of the C=O group at 1,723 cm^−1^, bands arising from symmetric vibrational vibrations of the CH_3_ group at 1,382 cm^−1^, a band from scissor vibrations of the CH_2_ group at 1,453 cm^−1^, peaks from asymmetric and symmetric stretching vibrations of the C–O–C ester group at 1,180 and 1,132 cm^−1^, and the peak at 1,230 cm^−1^ characteristic of the helical conformation of crystalline PHB were identified in the spectra (Xu et al., 2002). In addition, chitosan bands from C–O stretching vibrations (amide I) at 1,650 cm^−1^ and a band arising from amide II N–H stretching vibrations at 1,570 cm^−1^, which strongly overlap with the intense peaks of gelatin amide I (approximately 1,657–1,640 cm^−1^) and amide II (1,570–1,530 cm^−1^) vibrations (Hassan et al., 2021; Chys et al., 2011) (verified by the increase in intensity of these bands in the AGPCHLY spectrum), were found in both spectra.

Other characteristic bands arise from asymmetric C–H bending vibrations of the CH_2_ group (bands between 1,470–1,380 cm^−1^) (overlapping with CH_2_ and C–C bending vibrations of agarose), O-bridge stretching vibrations of glucosamine groups (at approximately 1,030 cm^−1^) of the chitosan chain, and the C–O–C stretching vibrations of the glycosidic linking between 1,170 and 1,050 cm^−1^ from agarose (Kumirska et al., 2010; Ali et al., 2021), but these bands only affected the shape of individual peaks of PHB in the spectra due to the high intensity and sharpness of the PHB vibrational modes. Note that an insignificant interaction between the PCHLY biopolymer components and the gel components was revealed after adding the gel to the PHB/chitosan scaffold. This can be explained, first, by the low content of agarose and gelatin in the gel, where their activity was affected by the high concentration of water molecules after swelling, and second, the interaction proceeded only with the solid surfaces of precipitated and dry (lyophilized) biopolymer fibers in the PCHLY scaffold. In addition, it is possible to consider an increased interaction between the hydrophilic biopolymers chitosan–agarose–gelatin during sterilization at 121°C, when the structure of the agarose/gelatin gel is destroyed and the gel transforms into a sol with the subsequent diffusion of water molecules between the chitosan fibers with their partial swelling. We believe that, despite this, the internal structure of the chitosan fiber remains too dense and unsuitable for the diffusion of larger macromolecules such as gelatin or agarose between chitosan fibers. Therefore, a stronger mutual interaction between the above components at the molecular level, especially in the bulk of the scaffold, is unlikely. The reaction between the hydroxyl groups of agarose and the amine/amide groups in chitosan or the formation of hydrogen bonds was indicated in films prepared from homogeneous mixtures of agarose and chitosan gels (Vivcharenko et al., 2020). On the other hand, in a homogeneous gel, no physicochemical interaction between agarose and gelatin in the gel was detected, and a two-phase system was identified depending on the content of the individual components (Almrhag et al., 2013).

### 3.2 Contact cytotoxicity test

**FIGURE 5:**
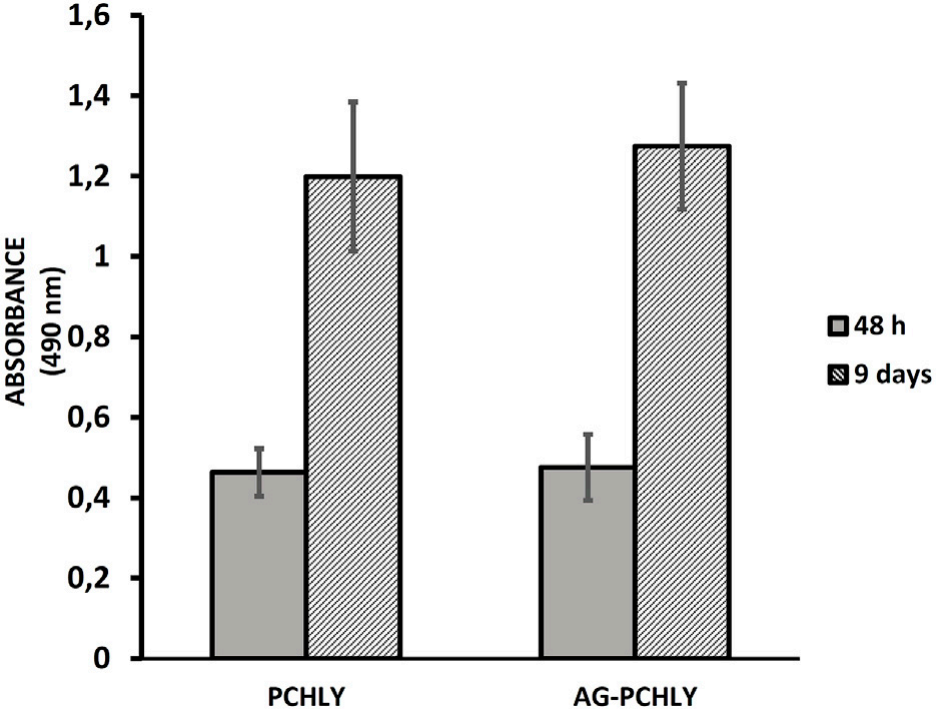
Proliferation of L929 fibroblasts after 48 h and 9 days of cultivation on polymeric scaffolds.

The results of *in vitro* contact cytotoxicity determination are shown in Figure 5. The measured absorbances (490 nm) of formazan in wells with the tested polymers rose with time and were double to triple after 9 days compared to measured absorbances after 48 h of culturing, but no statistically significant differences (p > 0.55) between proliferation of cells on both substrates were revealed. When comparing the differences in absorbance between days 2 and 9 of cultivation, statistically significant differences (p < 0.001) were observed in both types of substrates.

### 3.3 Live/dead staining

**FIGURE 6:**
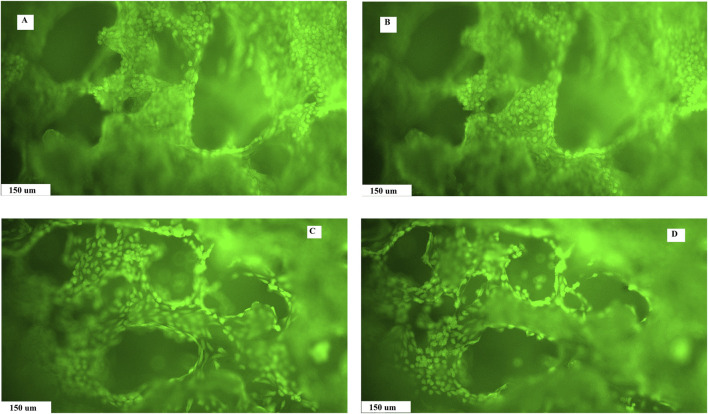
Live/dead staining of fibroblast growth on the surface and pore walls of PCHLY **(A, B)** and AG-PCHLY **(C, D)** scaffolds for 9 days in the culture medium (37°C, 95% humidity, and 5% CO_2_) observed in different depths of the substrates.

The live/dead staining of fibroblasts growing on the surface of the substrates and inside the macropore walls in a dense layer confirmed the non-cytotoxic nature of the composite biopolymer scaffolds, regardless of composition. Photomicrographs document the formation of a homogeneous uniform cell coating on substrates and the presence of cells in macropores, which clearly demonstrates the suitable open microstructure of the composites for cell migration into the internal structure of the scaffolds. The cells were well-adhered and spread out on substrates, and no dead cells (red colored) were identified on the layer. In addition, a reduction in size of macropores in AG-PCHLY scaffolds (Figures 6C, D) can be seen in images compared to that in PCHLY scaffolds (Figures 6A, B), which is consistent with the above results.

### 3.4 *In vivo* CAM assay

#### 3.4.1 Macroscopic evidence of angiogenic response

**FIGURE 7:**
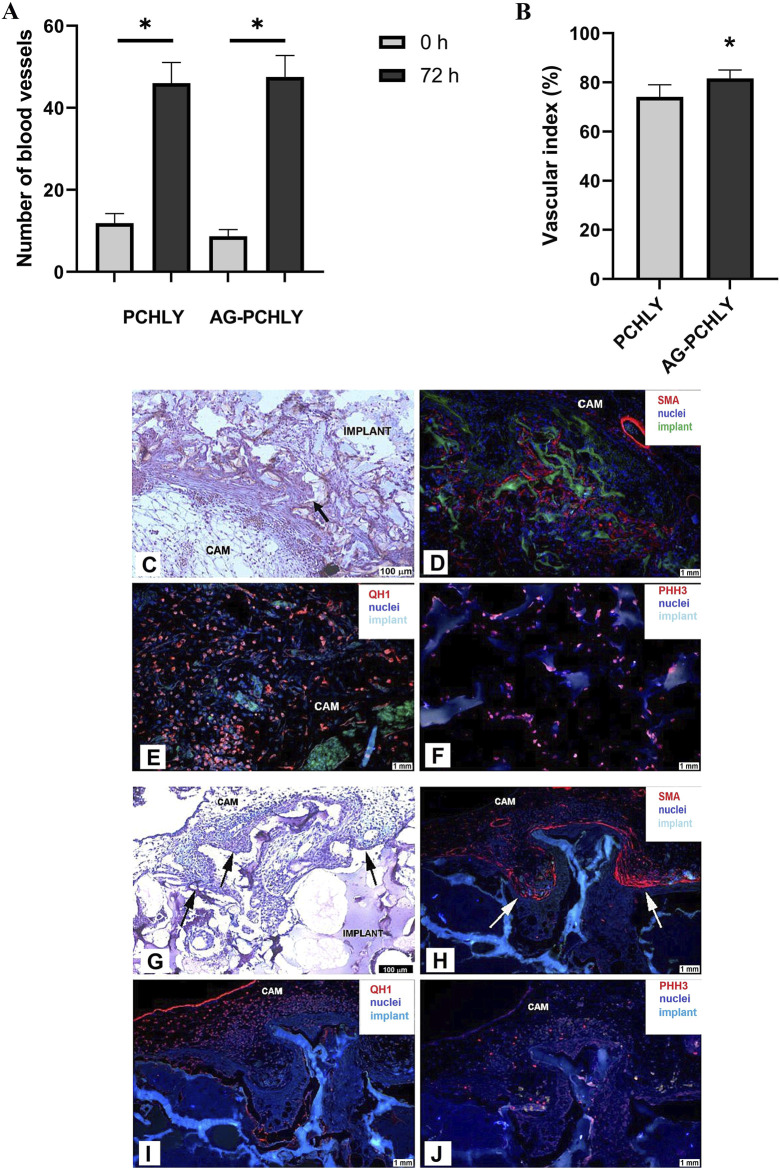
**(A)** Comparison of the average number of blood vessels in the surrounding area of the scaffolds on the day of scaffold implantation (ED6, 0h) and 72 h after implantation (ED9, 72 h). Experiments were repeated three times, and data are shown as mean ± SD (PCHLY: n = 8, AG-PCHLY: n = 10; technical replicates: two images per each biological replicate, one after the implantation and one 72 h after implantation). *p < 0.0001. **(B)** Vascular index as evidence of angiogenic response and *in vivo* angiogenic activity of the tested PCHLY and AG-PCHLY materials. Comparison of the average number of newly formed blood vessels in the surrounding area of the scaffolds. Experiments were repeated three times, and data are shown as mean ± SD (PCHLY: n = 8, AG-PCHLY: n = 10; technical replicates: two images per each biological replicate, one after the implantation and one 72 h after implantation). *p < 0.01. **(C–F)** Histological and immunohistochemical detection of biocompatibility and angiogenic potential of the PCHLY scaffold. **(C)** Formation of microvilli of the surrounding CAM tissue; scale bar: 100 μm, staining H–E. **(D)** Evidence of myofibroblasts within the CAM tissue microvilli; scale bar: 1 mm, α-SMA marker. **(E)** Detection of hemangioblasts in both the surrounding CAM tissue and microvilli; scale bar: 1 mm, QH1 marker. **(F)** Visualization of proliferating cells in the surrounding CAM tissue; scale bar: 1 mm, PHH3 marker; CAM: chorioallantoic membrane; arrow: CAM microvilli. **(G–J)**: Histological and immunohistochemical detection of biocompatibility and angiogenic potential of the AG-PCHLY scaffold. **(G)** Formation of microvilli of the surrounding CAM tissue; scale bar: 100 μm, staining H–E. **(H)** Evidence of myofibroblasts within the CAM tissue microvilli; scale bar: 1 mm, α-SMA marker. **(I)** Detection of hemangioblasts in the surrounding CAM tissue; scale bar: 1 mm, QH1 marker. **(J)** Visualization of proliferating cells in both the surrounding CAM tissue and microvilli; scale bar: 1 mm, PHH3 marker; CAM: chorioallantoic membrane; arrow: CAM microvilli.

The evaluation of *in vivo* angiogenic activity of the tested scaffolds showed angiogenic potential in both materials. However, the vascular index evaluation confirmed the higher angiogenic response of AG-PCHLY (81.59% ± 3.42%) compared to that of PCHLY (74.12% ± 4.90%) 72 h after implantation (Figures 7A, B).

#### 3.4.2 Histological and immunohistochemical analyses

Microscopic evaluation of the angiogenic response of the CAM tissue to the implanted PCHLY and AG-PCHLY scaffolds was conducted on serial histological sections of the CAM/PCHLY and CAM/AG-PCHLY complexes. The formation of new CAM villi and their successful incorporation into the scaffold were observed in both samples, which suggests good biocompatibility of the scaffold (Figures 7C, G). Furthermore, confirmation of ongoing angiogenesis and ingrowth of newly formed blood vessels inside the scaffolds were accomplished by the identification of the specific markers associated with the process of angiogenesis and biocompatibility by immunohistochemical analysis.

The overexpression levels of smooth muscle alpha-actin (α-SMA) suggested the presence of activated fibrogenic cells, known as myofibroblasts, around both tested materials, the PCHLY (Figure 7D) and AG-PCHLY scaffolds (Figure 7H). The most pronounced positivity was observed in the periphery of the PCHLY scaffold compared to the CAM microvilli location in the AG-PCHLY material. Quail endothelial and hemopoietic cells were detected using the quail-specific immunomarker (QH1), which is used for the detection of quail endothelial cells. We observed the presence of individual endothelial cells mostly in the surrounding CAM tissue of the AG-PCHLY scaffold (Figure 7I) compared to that of PCHLY (Figure 7E), where we also observed the presence of QH1-positive cells inside the implanted scaffold. Detection of QH1-positive cells confirmed the presence of a vascular network on the surface of the implants as a sign of ongoing neovascularization. Phosphohistone H3 (PHH3) is the conventional proliferative marker for cells undergoing mitosis. Using the immunohistochemical analyses of anti-PHH3, we assessed the mitotic activity of the cells in the evaluated scaffolds. In AG-PCHLY (Figure 7J), we observed a higher mitotic activity around the implant contrary to PCHLY, where the mitotic activity was seen in the surrounding CAM tissue and inside the pores (Figure 7F).

The immunohistochemical staining and the detection of the mentioned immunomarkers (α-SMA, QH1, and PHH3) provide the evidence of biocompatible and angiogenic properties of the tested scaffolds.

#### 3.4.3 Gene expression analysis

**FIGURE 8:**
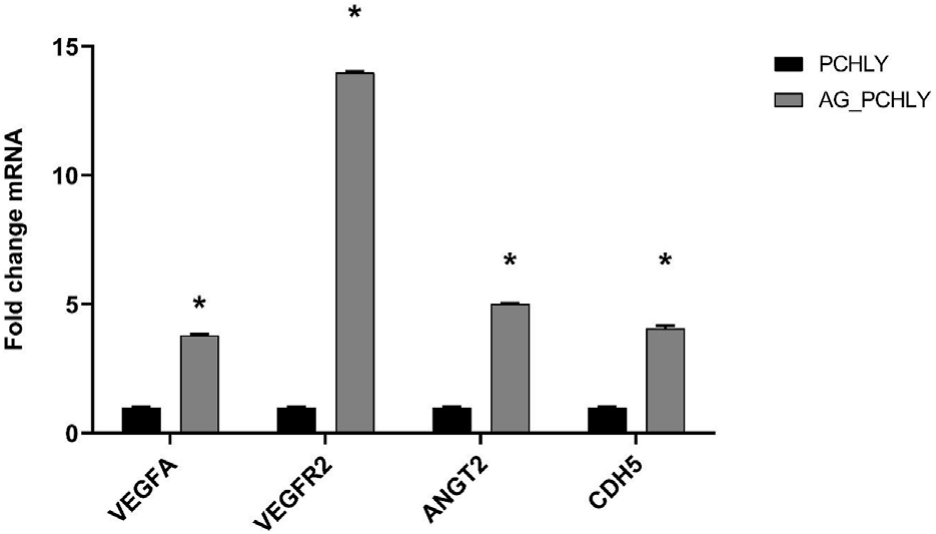
qRT-PCR analysis of gene expression.

The qRT-PCR analysis of gene expression (Figure 8) clearly demonstrated significant upregulation of all studied genes (*VEGFA*, *VEGFR2*, *ANGT2*, and *CDH5)* in AG-PCHLY biomaterial (p < 0.0001) than in the control PCHLY biomaterial. The increased potential pro-angiogenic effect of AG-PCHLY gel-containing biopolymer mixtures compared to that of PCHLY was verified by higher transcription of the VEGFA gene, which promotes higher expression of VEGFR2 on the endothelial cell. Angiopoietin 2, synergistically with VEGFA, promotes the growth of new blood vessels, and VE-cadherin is involved in the formation of adhesive junctions between endothelial cells. For the statistical analysis, two-way ANOVA was used with the Sidak test (p < 0.0001).

## 4 Discussion

Polymeric blends that consisted of various combinations of chitosan, PHB, agarose, and gelatin, respectively, were successfully used in tissue engineering techniques.

A chitosan–agarose–gelatin scaffold fabricated by cryogelation crosslinked with glutaraldehyde resulted in the formation of an interconnected elastic porous network that can be utilized in skin and cardiac tissue engineering (Bhat and Kumar, 2012). Felfel et al. studied the correlation between mechanical and structural properties of chitosan–agarose (Ch–Agrs) blends, and the results showed that increasing the agarose content in the blend led to a reduction in pore size, significant increase in the compressive strength, and improved stability of the blends in aqueous media (Felfel et al., 2019). Despite different preparation procedures, the findings of Felfel et al. agree with our results, confirming that the addition of agarose/gelatin gel improved the mechanical stability of PHB–chitosan scaffolds.

Angiogenesis, as a process of growth of new vessels from pre-existing vessels, and it is essential for the acceptance of implanted xenografts by human and animal bodies in regenerative medicine. A functional vascular network built within the engineered scaffold is required to provide a functional environment for new tissue formation, ingrowth, and maturation. For enhancement of vascularization and angiogenesis, various approaches have been made by introducing angiogenic factors and molecules to different scaffolds and hydrogels. Chitosan/hyaluronic acid/VEGF-loaded nano fibrin composite sponges were developed with the potential to induce angiogenesis in wound healing (Mohandas et al., 2015). The VEGF/chitosan nanoparticles showed a protective effect on vascular endothelial cells, enhanced local microcirculation, and improved skin healing after radiation-induced skin injury (Yu et al., 2016).

A tri-layered fibrous scaffold incorporating VEGF and platelet factor concentrate consisting of poly(hydroxybutyrate-co-hydroxyvalerate) (PHBV) and poly(vinylalcohol) (PVA) nanofibers was designed to mimic the native arteries (Deepthi et al., 2018). An injectable, biodegradable chitosan–fibrin (CF)-based hydrogel was developed, in which vascular endothelial cells seeded in the CF hydrogel were able to form capillary-like structures (Hsieh et al., 2017).

In our previous paper, a cheap and simple acellular chitosan/polyhydroxybutyrate porous scaffold was tested in CAM assay, and strong angiogenic potential was found with the formation of the CAM villi and the presence of endothelial cells in new blood vessels in pores of scaffolds. However, despite the positive biological characteristics, the biomaterial had lower mechanical strength after wetting, which is not an advantageous property for application (Demcisakova et al., 2022). The composition of the above-mentioned scaffold was modified by gel without the addition of expensive and relatively low-stability growth factors or cytokines, but this change could significantly affect the angiogenic properties of the scaffolds. From the results of the quail CAM assay, it is evident that porosity and pore size diameter strongly influence the ingrowth of vessels into deeper sites of implants.

The porosity of implants allows the migration of the cells into the scaffolds and ensures the transport of cellular nutrition and waste products. Physical and electrochemical surface characteristics of the biomaterial (chemistry, wettability, stiffness, roughness and topography, interconnectivity, pore morphology, and orientation) greatly influence cell adhesion, infiltration, and differentiation properties and the biological response in *in vitro* or *in vivo* environments. Sufficient porosity is essential for the settlement of cells in scaffolds, as well as their good attachment, proliferation, and differentiation, which leads to increased tissue ingrowth. High and uniform pore interconnectivity in a 3D scaffold is crucial for diffusion and the exchange of nutrients and metabolites between the scaffold and the surrounding. Furthermore, adequate mechanical stability and degradation rate are necessary to ensure a suitable environment for cells to attach and form their own ECM and reduce the deformation or failure of the scaffolds (Ellermann et al., 2023).

Analysis of our data showed that ∼50% pores (AG-PCHLY) and ∼60% of pores (PCHLY) in the scaffolds were smaller than 100 µm. A total of 20% of pores had a diameter between 100–200 µm. Only 2% of pores showed a pore diameter of 400 μm but only in case of the PCHLY scaffold. Despite this, we note that in terms of volume fraction and using, for example, the spherical approximation, the pore volume increases with the third power of the diameter so that even a small fraction of larger pores will have a significant volume fraction in the pore microstructure of the scaffold. Thus, the presence of individual endothelial cells mostly in the surrounding CAM tissue of the AG-PCHLY scaffold compared to that of the PCHLY scaffold, where the endothelial cells were visible inside the implanted scaffold, was due to pores with a larger pore diameter. In addition, a vascular network was identified on the surfaces of both types of biopolymer implants as a sign of neovascularization. Similarly, the AG-PCHLY scaffold showed a higher mitotic activity of cells around the implant than the PCHLY scaffold with the mitotic activity of cells in the surrounding CAM tissue and inside the material pores. However, despite the above results, the number of blood vessels and vascular index 72 h after scaffold implantation indicated better pro-angiogenic properties of AG-PCHLY scaffolds; thus, the agarose/gelatin gel significantly promoted angiogenesis.

Mastrullo et al. (2020) published that the minimum overall porosity of approximately 50%, along with a pore size of approximately 35–100 μm, is considered optimal for blood vessel formation (Mastrullo et al., 2020), but on the contrary, Samourides et al. revealed that poly-(glycerol sebacate urethane) scaffolds with a pore diameter of only 26–28 µm showed good conditions for cell adhesion and pro-angiogenic properties in avian CAM assay (Samourides et al., 2020). Chiu et al. examined poly-ethylene glycol (PEG) hydrogels, where the cell and vessel invasion into gels with 25–50-µm sized pores was limited to the external surface only and gels with larger pores (50–100 and 100–150 µm) permitted vascularization throughout the entire material volume (Chiu et al., 2011). It has been shown that biomaterials with pore sizes >100 μm support bone formation, where larger pores over 500 μm and pores with a large surface area and interconnectivity are associated with enhanced vascularization and bone formation *in vivo* (Eichholz et al., 2022).

The evaluated biocompatible PHB/chitosan scaffolds with added agarose/gelatin gel exhibited suitable microstructure, porosity, and other properties necessary for promoting angiogenesis. These scaffolds show promise as biomaterials for cartilage tissue engineering (Giretova et al., 2019) and wound dressing applications (Humenik et al., 2024).

## 5 Conclusion

The biopolymeric composite PHB/chitosan scaffolds with the agarose/gelatin gel were prepared and evaluated for their basic material characteristics. The gel addition supported structural and mechanical stabilization of macroporous scaffolds without any tendency to disintegrate, break, or crumble after soaking in cell culture media for 9 days. *In vitro* testing showed no sign of cytotoxicity, enhancement of cells ingrowth, and pro-angiogenic potential in CAM test, as well as an upregulation of genes that promote angiogenesis, which predispose these scaffolds for utilization in the field of regenerative medicine. Note that the prepared acellular scaffolds represent a simple and relative cheap biomaterial without additives such as growth factors or other bioactive molecules, which are often used to improve angiogenesis after implantation into the body. The abovementioned features predetermine the possible future utilization of these natural biopolymeric scaffolds, mainly in the area of cartilage and skin regenerative medicine.

## Data Availability

The original contributions presented in the study are included in the article/supplementary material; further inquiries can be directed to the corresponding author.
